# The Description of Shale Reservoir Pore Structure Based on Method of Moments Estimation

**DOI:** 10.1371/journal.pone.0151631

**Published:** 2016-03-18

**Authors:** Wenjie Li, Changcheng Wang, Zejin Shi, Yi Wei, Huailai Zhou, Kun Deng

**Affiliations:** 1 College of Energy Resources, Chengdu University of Technology, Chengdu, China; 2 State Key Laboratory of Oil and Gas Reservoir Geology and Exploitation, Chengdu University of Technology, Chengdu, China; 3 Research Institute of Exploration and Development, Daqing Oil Field Company, Daqing, China; 4 College of Geophysics, Chengdu University of Technology, Chengdu, China; College of Agricultural Sciences, UNITED STATES

## Abstract

Shale has been considered as good gas reservoir due to its abundant interior nanoscale pores. Thus, the study of the pore structure of shale is of great significance for the evaluation and development of shale oil and gas. To date, the most widely used approaches for studying the shale pore structure include image analysis, radiation and fluid invasion methods. The detailed pore structures can be studied intuitively by image analysis and radiation methods, but the results obtained are quite sensitive to sample preparation, equipment performance and experimental operation. In contrast, the fluid invasion method can be used to obtain information on pore size distribution and pore structure, but the relative simple parameters derived cannot be used to evaluate the pore structure of shale comprehensively and quantitatively. To characterize the nanoscale pore structure of shale reservoir more effectively and expand the current research techniques, we proposed a new method based on gas adsorption experimental data and the method of moments to describe the pore structure parameters of shale reservoir. Combined with the geological mixture empirical distribution and the method of moments estimation principle, the new method calculates the characteristic parameters of shale, including the mean pore size ($\bar{x}$), standard deviation (*σ*), skewness (*S*_*k*_) and variation coefficient (*c*). These values are found by reconstructing the grouping intervals of observation values and optimizing algorithms for eigenvalues. This approach assures a more effective description of the characteristics of nanoscale pore structures. Finally, the new method has been applied to analyze the Yanchang shale in the Ordos Basin (China) and Longmaxi shale from the Sichuan Basin (China). The results obtained well reveal the pore characteristics of shale, indicating the feasibility of this new method in the study of the pore structure of shale reservoir.

## 1. Introduction

Shale has attracted increasing attention recently due to its nanoscale pores containing abundant oil and gas resources and has become a focus of global unconventional oil and gas exploration and development. Compared to conventional reservoirs, shale is a tight reservoir with a complex origin and strong heterogeneity [1–2]. Their pores have a complex geometry at the nanoscale and have low porosity and ultra-low permeability [3–6]. Previous studies have investigated the pore size and distribution of different shale formations. The Barnett shale in North America has various types of pores [6–8], with an organic nanopore distribution between 5 and 750 nm, a median size of approximately 100 nm [6]. The pore size of the marine shale in South China is between 2 and 900 nm, with an average of approximately 3 to 40 nm [9–14]. The pores of the continental shale of North China have a distribution between 2 and 35 nm [15–18]. The abundant nanoscale pores of shale provide good spaces for gas accumulation, playing an important role for gas storage and migration [1,6,8,19–21]. Thus, the study of pore structure features is of great significance for shale oil and gas evaluation as well as for exploration and development.

Pore structure refers to the geometry, size, distribution and the interconnected relationships between the pores and throats in rocks [22]. In recent years, transmission/scanning electron microscopy (TEM/SEM), field emission scanning electron microscopy (FE-SEM), atomic-force microscope (AFM), nuclear magnetic resonance (NMR), small-angle and ultra-small-angle neutron scattering (SANS and USANS), X-ray micro tomography (XMT), mercury injection capillary pressure (MICP) and gas adsorption have been used to study the pores of shale [3–8,20,23–28]. Image analysis methods can be used to observe the pores developed in rocks through TEM/SEM, FE-SEM and other high resolution microscopies. The obtained image data can be used to analyze the geometry and connectivity of pores. Although a large quantity of pore throat information can be acquired intuitively using image analysis, the results obtained depend on the device resolution, and a large number of experiments are needed due to the small imaging scope. Moreover, it is difficult to conduct quantitative analyses with image analysis. NMR, SANS, Nano-CT and other radiation methods be used to conduct non-destructive analysis and acquire detailed data rapidly, but the measurement errors are strongly influenced by sample preparation and heterogeneity. In addition, radioactive sources are rare and quite expensive. Researchers often use a fluid invasion method (e.g., MICP, N_2_ adsorption, or CO_2_ adsorption) to determine the shale pore size distribution and use theoretical models to calculate the specific surface area, the volume and other parameters.

In the gas adsorption method, the volume of liquid nitrogen filled in test samples is an equivalent volume of the pore space under isothermal conditions. A relationship between relative pressure (*P/P*_*0*_) and gas absorption (desorption) volume (*V*) can be constructed by measuring gas adsorption volumes under different relative pressures (*P/P*_*0*_). The resulting adsorption and desorption isotherms can be used to obtain the specific surface area and pore size distribution using theoretical model calculations [29]. At present, commonly used theoretical models include Barrett-Joyner-Halenda (BJH) [30], Horváth-Kawazoe (HK) [31], density functional theory (DFT) [32] and quenched solid density functional theory (QSDFT) [33–34] models. According to the pore size classification standard of the International Union of Pure and Applied Chemistry (IUPAC), the three categories of pores are micropores (diameter < 2 nm), mesopores (2 nm < diameter < 50 nm) and macropores (diameter > 50 nm) [35]. Of the models mentioned above, the BJH model can effectively measure the mesopores with pore sizes of 2 to 50 nm [30,36]. The HK model is commonly used to analyze microporous materials [31]. The DFT model is based on the Tarazona equation and QSDFT model, which improves upon the DFT model and can be used to effectively analyze microporous–mesoporous materials [32–34]. Although it has been extensively used to characterize the pore structure of shale, the gas adsorption method still has some drawbacks: e.g., it requires complex theoretical model calculations, and the results obtained by different models show larger errors [37–38].

Theoretically, the gas adsorption method can measure open pores with a diameter larger than 0.35 nm, which is the diameter of nitrogen molecules. However, only pores with a diameter between 1.5 and 200 nm can be measured effectively due to the limitations of instrument accuracy and experimental operation difficulty. Furthermore, the parameters obtained by the gas adsorption method are relative simple and cannot be used to evaluate the mean pore size, pore sorting and pore structure comprehensively and quantitatively.

The major goal of this paper is to proposed a new method to describe the pore structure parameters of shale reservoir. The new method is based on gas adsorption experimental data and, combined with the geological mixture empirical distribution and the method of moments estimation principle, optimizes eigenvalue algorithms and determines pore structure parameters by applying the method of moments. Using this method, the calculation of the parameters of the pore structure is simplified, and the nanopores in shale can be described. This study aims to expand the current methods for evaluating the pore structure of shale, and simplify the algorithm of eigenvalues.

## 2. Method Description

Based on extensive investigations Luo and Wang (1981) discovered that the pore throat distribution of limestone and carbonate rocks did not comply with the normal distribution proposed by Chilingar (1972). Instead, the distribution was a combination of various pore throat distributions, which should be caused by multiple factors of diagenesis and epidiagenesis. For a mixed distribution, the eigenvalues can be determined by the method of moments according to the numerical characteristics of the empirical geological distribution [22]. Using the method of moments, Luo (1981) and Wang (2003) extensively studied the low permeability reservoirs of the oil and gas bearing basins in China, such as the Ordos Basin, Sichuan Basin, and Bohai Bay Basin. The characteristic parameters obtained revealed the pore structure characteristics of reservoir rocks and provided constructive guidance for the exploration and development of oil and gas fields [22,39].

The obtained date should be grouped before the method of moments is used to calculate the characteristic parameters of pore structure. The shale reservoirs with low permeability, low porosity of nanoscale pores belong to the tight-ultra-tight reservoirs [3–6]. Thus, the grouping interval of the observation values of sandstone and carbonate rocks is not suitable for the grouping of shale observation values, and a new grouping observation interval is required. By taking the gas adsorption volumes (under liquid conditions) during adsorption experiments as the observation values (equivalent to saturation), the functional relationship between relative pressure (*P/P*_*0*_) and mean pore size (*Φ*) can be established by the classical Kelvin equation [40]

rk=−0.414log(P/P0) (1)

Halsey equation [29]

t=0.354[−5/ln(P/P0)]13 (2)

$$D=2\left(r_{k}+t\right)$$ (3)

and conversion equation [22]

$$\Phi =—log_{2}{}^{D}$$ (4)

where *r*_*k*_ is the capillary radius in nm, *P/P*_*0*_ is the relative pressure, *t* is the multilayer thickness in nm, *r*_*p*_ is the pore radius in nm and *D* is the pore size (diameter) in nm in Eq (3) and expressed by mm in Eq (4).

According to the Oil and Gas Industry Standard of People's Republic of China [41] and the National Standard of the People's Republic of China [42] for gas adsorption, the relative pressure range (*P/P*_*0*_ = 0.250–0.995) determines the scope of *Φ* value: 11.35–18.35*Φ*. With equal spacing of *Φ*, the observation values can be divided into fifteen intervals with a width setting of 0.5*Φ*. This classification method actually shortens the coarse pore part intervals, widens the fine pore part intervals and considers the proportion of different magnitude pore weights. As such, the calculated characteristic parameters reveal the pore characteristics of shale well. The method of moments can analyze and evaluate the pores more effectively with the refined interval.

The observation values can be summarized as a mathematical process using mathematical language. For many types of observation values, important mathematical characteristic parameters of rock pore throats include:

1. Mean value ($\bar{x}$)
  The mean is one of the parameters to describe the centralized location of data; it describes the average position of experimental data. It represents the average value of the entire pore size distribution for the pore structure of reservoir rocks. This value can be acquired through the weighted average of the observation values in our study, namely
  x¯=∑i=1nxifiSmax (5)
  where *x*_*i*_ is the start value (mid-value or end-value) of the i-th interval, which can be expressed by *Φ* for reservoir rocks. A greater *Φ* value indicates a smaller pore size (*D*). *f*_*i*_ is the observation value (Δ*S*_*i*_), i.e., the nitrogen saturation under liquid conditions that can be expressed as a percentage, *S*_*max*_ is the maximum inlet nitrogen saturation and *n* is the number of groups with a default value of 15. This algorithm accounts for the proportion of pores of different sizes according to the weighted data and is more in line with the realistic situation of pore size distribution.
2. Standard deviation (*σ*)
  The standard deviation is the scattered characteristic parameter. It is the mean-centered measure of the degree of dispersion of the data. It describes the degree of dispersion of the experimental data over the entire number axis and represents the extent of dispersion from the mean value. In this paper, the standard deviation refers to the degree of dispersion of pore size with respect to the mean and can also be called the sorting coefficient of the pores. For the pore system, a smaller standard deviation of pore indicates a better sorting coefficient.
  σ=[∑i=1nfi(xi−x¯)2Smax]12 (6)
3. Variation coefficient (*c*)
  The variation coefficient, defined as the ratio of the standard deviation to the mean value, is a useful measurement for the variation of observation values. It can be applied to describe the ratio between the mean pore size (*Φ* value) and sorting degree. If the mean pore size (*Φ* value) is large (more fine pores) and the sorting degree is good (a smaller degree of dispersion for the pores), the value of *c* will be small. Within a certain range, *c* values can be used to evaluate the quality of the reservoir pore structure. In general, a large value of *c* indicates a more discrete degree of the pores. A large difference in the pores size is conducive to gas storage and migration, indicating a good pore structure of the reservoir rock.
  c=σx¯ (7)
4. Skewness (*S*_*k*_)
  The skewness is a measurement of the direction and extent of the data distribution. In geology, skewness reflects the pore size distribution; it describes the inclination to a larger or smaller size from mean value, with a coarse skewness indicating larger pores, and vice versa.
  Sk=∑i=1nfi(xi−x¯)3σ3.Smax (8)

In [22] Luo treated the *S*_*max*_ in Eqs (5), (6) and (8) as a constant of 100 by regarding the maximum mercury saturation as 100% during the MICP. Li (2001) suggested the calculated results would be overestimated when taking the *S*_*max*_ as a constant 100, which is impractical [43]. Noticed that shale has low porosity and ultra-low permeability and that closed pores may exist within shale. Therefore, pores may not always be fully filled by nitrogen, and saturation may not reach 100%. As such, in our method, *S*_*max*_ was introduced into the denominator of Eqs (5), (6) and (8) as a variable.

## 3. Experimental Methods and Data Processing

### 3.1. Samples and experiments

Twenty-two samples (Y-1 to Y-22) were collected from the Triassic Yanchang shale in the southern area of the Ordos Basin (China), and fifteen samples (Y-23 to Y-37) were collected from the Silurian Longmaxi shale in the southeast area of the Sichuan Basin (China). Nitrogen adsorption was conducted in the State Key Laboratory of Oil and Gas Reservoir Geology and Exploitation of Chengdu University of Technology, Chengdu, China. The QUADRASORB SI specific surface and porosity analyzer was used to test the thirty-seven shale samples and obtain the nitrogen adsorption data.

The relative pressure range of the instrument is 0.005–0.995, and the measuring range is 0.35–400 nm. The samples crushed to less than 0.250 mm. Before analysis, every sample should be degassed by vacuum to ensure that adsorbed moisture and volatile matter were removed. In our study, samples were dried under a vacuum at 80°C for at least 22 h. After drying, samples underwent the nitrogen adsorption test under the relative pressure range from 0.005–0.995 at 77.3K.

### 3.2. Data processing

**Fig 1:**
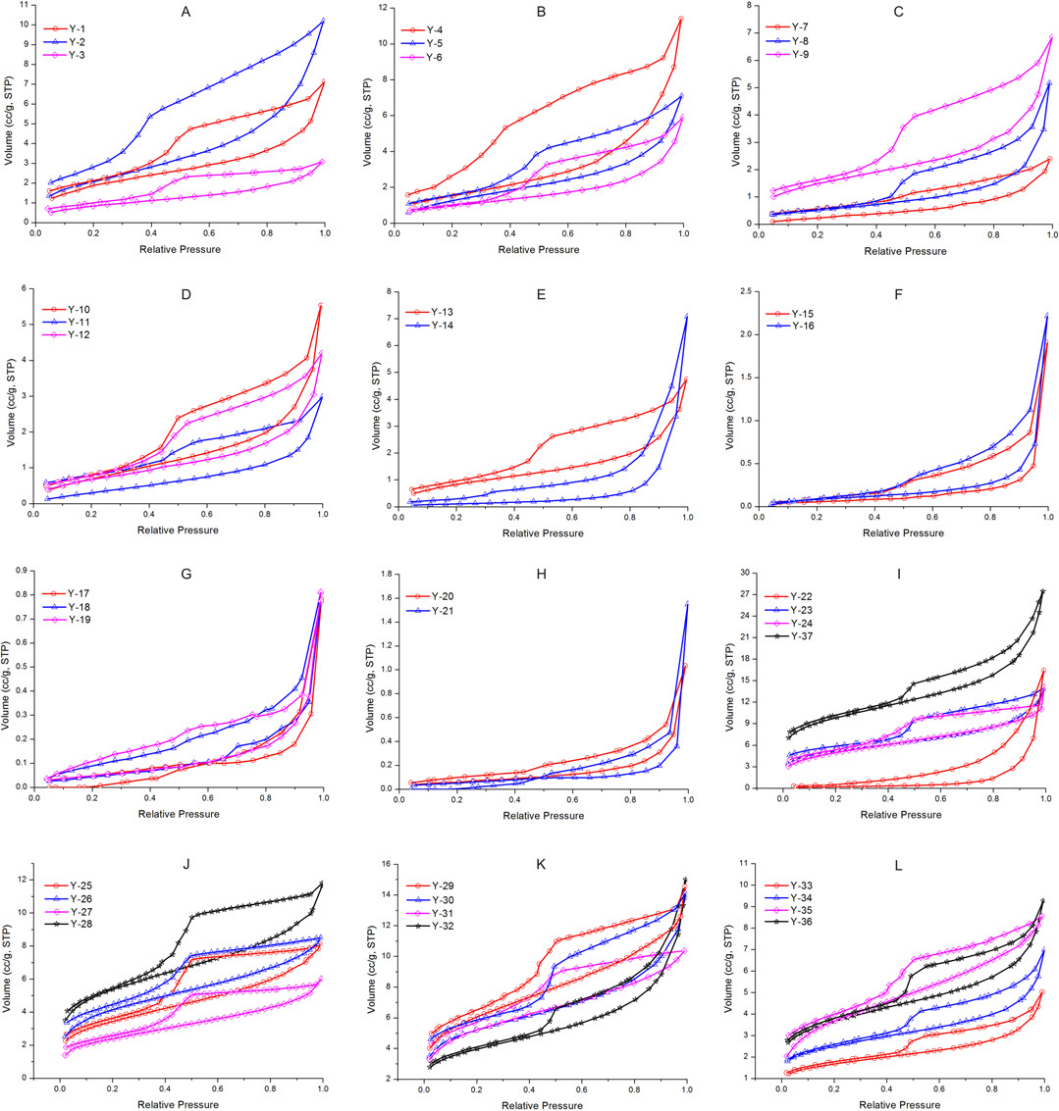
Nitrogen adsorption and desorption isotherms for the thirty-seven shale samples at 77.3K. Samples Y-1 to Y-22 were collected from Triassic Yanchang shale in the southern area of the Ordos Basin, and samples Y-23 to Y-37 from Silurian Longmaxi shale in the southeast area of the Sichuan Basin, China.

Sample Y-1 illustrates the data processing process. First, an adsorption-desorption curve was plotted (Fig 1A) from the original data from the nitrogen adsorption experiment (Table 1). The nitrogen desorption volumes *V*_*i*_(*g*) under different relative pressures were extracted from the desorption branch and then converted into liquid volumes *V*_*i*_(*l*) to calculate the inlet nitrogen saturation (*S*_*i*_) and interval saturation (Δ*S*_*i*_). Finally, the characteristic parameters, such as mean pore size ($\bar{x}$), standard deviation (*σ*), variation coefficient (*c*) and skewness (*S*_*k*_) can be calculated by substituting *S*_*i*_ and Δ*S*_*i*_ into Eqs (5–8), as shown in Table 2. The experimental data of the other thirty-six shale samples were processed by the above method, and the results are shown in Table 3.

**Table 1:** Original data of the nitrogen adsorption experiment for sample Y-1.

Relative pressure	Volume @ STP [cc/g]	Relative pressure	Volume @ STP [cc/g]	Relative pressure	Volume @ STP [cc/g]
5.61109e-02	1.2307	6.99139e-01	3.2049	6.32688e-01	5.1002
9.67624e-02	1.4402	7.48229e-01	3.3829	5.84258e-01	4.9229
1.46011e-01	1.6354	7.98605e-01	3.6523	5.35243e-01	4.7384
1.96100e-01	1.8720	8.54699e-01	4.0014	4.90213e-01	4.2285
2.47011e-01	2.0061	9.23365e-01	4.6588	4.51873e-01	3.5208
2.95116e-01	2.1251	9.50960e-01	5.1378	3.98044e-01	3.0267
3.48446e-01	2.2806	9.99001e-01	7.1283	3.43955e-01	2.6921
3.97044e-01	2.4024	9.42076e-01	6.2607	2.87444e-01	2.4391
4.45840e-01	2.5172	8.75939e-01	5.9450	2.36163e-01	2.2469
4.96021e-01	2.6331	8.23074e-01	5.7397	1.86130e-01	2.0819
5.45999e-01	2.7489	7.79427e-01	5.5852	1.32958e-01	1.9181
5.95547e-01	2.9156	7.32621e-01	5.4347	8.21741e-02	1.7514
6.49458e-01	3.0516	6.83317e-01	5.2762	4.83732e-02	1.6022

**Table 2:** Eigenvalues calculation table processed by the method of moments for sample Y-1.

*P/P*_*0*_	*x*_*i*_(*Φ*)	*V*_*i*_(*g*)	*V*_*i*_(*l*)	*S*_*i*_(*%*)^a^	Δ*S*_*i*_(*%*)	*x*_*i*_*·*Δ*S*	(*x*_*i*_-$\bar{x}$)^2^*·*Δ*S*	(*x*_*i*_-$\bar{x}$)^3^*·*Δ*S*
0.9950	11.35	7.0867	0.010984	99.42	0.73	8.28	24.96	-146.04
0.9928	11.85	7.0347	0.010904	98.69	0.43	5.14	12.41	-66.38
0.9898	12.35	7.0038	0.010856	98.25	1.06	13.15	25.05	-121.47
0.9855	12.85	6.9279	0.010738	97.19	1.25	16.01	23.57	-102.54
0.9794	13.35	6.8391	0.010601	95.94	1.85	24.66	27.39	-105.43
0.9706	13.85	6.7074	0.010396	94.10	3.00	41.58	33.69	-112.87
0.9580	14.35	6.4934	0.010065	91.09	3.26	46.82	26.50	-75.54
0.9401	14.85	6.2608	0.009704	87.83	1.85	27.52	10.23	-24.05
0.9142	15.35	6.1287	0.009499	85.98	2.54	38.93	8.68	-16.06
0.8770	15.85	5.9479	0.009219	83.44	2.92	46.29	5.32	-7.19
0.8225	16.35	5.7397	0.008897	80.52	3.34	54.68	2.42	-2.05
0.7505	16.85	5.5013	0.008527	77.18	4.87	82.14	0.60	-0.21
0.6475	17.35	5.1538	0.007988	72.30	9.19	159.40	0.21	0.03
0.5145	17.85	4.4989	0.006973	63.11	24.77	442.12	10.46	6.80
0.3500	18.35	2.7333	0.004237	38.34	38.34	703.62	50.71	58.32
Cumulative value						1710.36	262.21	-714.68
Eigenvalues				Mean value: $\bar{x}$ = ∑*x*_*i*_***·***Δ*S*/*S*_*max*_ = 17.20				
				Standard deviation: *σ* = [∑(*x*_*i*_-$\bar{x}$)^2^***·***Δ*S*/*S*_*max*_]^1/2^ = 1.62				
				Variation coefficient: *c* = *σ*/$\bar{x}$ = 0.094				
				Skewness: *S*_*k*_ = [∑(*x*_*i*_-$\bar{x}$)^3^***·***Δ*S*]/(*σ*^3^*S*_*max*_) = 1.691				

^a^ *S*_*i*_ = *V*_*i*_ (*l*)/*V*_*max*_(*l*)×100%, where *V*_*max*_(*l*) is total adsorption volume under liquid condition.

**Table 3:** Eigenvalues of thirty-seven shale samples calculated by the method of moments.

Sample ID	$\bar{x}$(*Φ*)	*D*_m_(nm)^a^	*σ*(*Φ*)	*c*(*Φ*)	*S*_*k*_(*Φ*)	*D*_bjh_(nm)^b^
Y-1	17.20	6.64	1.62	0.094	-1.691	5.66
Y-2	17.33	6.03	1.40	0.081	-1.645	5.34
Y-3	17.45	5.59	1.41	0.081	-1.930	4.54
Y-4	17.02	7.52	1.74	0.102	-1.237	7.23
Y-5	17.12	7.02	1.48	0.086	-1.374	5.95
Y-6	16.87	8.35	1.72	0.102	-1.278	5.73
Y-7	16.90	8.18	1.60	0.095	-1.041	6.09
Y-8	16.15	13.75	1.81	0.112	-0.035	7.71
Y-9	16.94	7.95	1.72	0.102	-1.313	6.25
Y-10	16.35	11.97	2.00	0.122	-0.704	6.70
Y-11	16.93	8.01	1.74	0.103	-1.176	6.35
Y-12	16.83	8.58	1.73	0.103	-1.207	6.25
Y-13	16.80	8.76	1.78	0.106	-1.119	6.49
Y-14	15.21	26.38	1.71	0.112	-0.029	22.39
Y-15	15.26	25.48	1.92	0.126	-0.076	13.70
Y-16	15.20	26.57	1.87	0.123	0.047	14.59
Y-17	15.09	28.67	1.60	0.106	0.302	15.21
Y-18	15.68	19.05	1.96	0.125	0.145	11.43
Y-19	15.70	18.79	1.80	0.096	0.078	10.49
Y-20	15.55	20.84	1.77	0.114	0.165	16.91
Y-21	14.66	38.63	1.74	0.119	0.273	23.07
Y-22	15.41	22.97	1.60	0.104	-0.042	15.08
Y-23	17.21	6.60	1.66	0.096	-1.692	6.04
Y-24	16.94	7.95	2.17	0.128	-1.381	5.81
Y-25	17.86	4.20	1.00	0.056	-3.444	3.92
Y-26	17.95	3.95	0.83	0.046	-3.386	4.03
Y-27	17.63	4.93	1.46	0.082	-2.026	4.43
Y-28	17.71	4.66	1.28	0.072	-2.863	4.38
Y-29	17.46	5.55	1.59	0.091	-2.052	5.01
Y-30	17.38	5.86	1.50	0.086	-1.925	5.46
Y-31	17.98	3.87	0.76	0.042	-3.258	4.24
Y-32	16.45	11.17	1.95	0.175	-0.698	8.49
Y-33	16.87	8.35	1.83	0.108	-1.005	6.80
Y-34	16.96	7.84	1.86	0.110	-1.256	5.95
Y-35	17.66	4.83	1.20	0.068	-2.213	4.78
Y-36	17.09	7.17	1.82	0.106	-1.419	8.49
Y-37	16.86	8.41	1.76	0.104	-0.871	8.01

^a^ *D*_m_ is the mean pore size (diameter) expressed by nm from the method of moments.

^b^ *D*_bjh_ is average pore diameter from the BJH method.

## 4. Results

**Fig 2:**
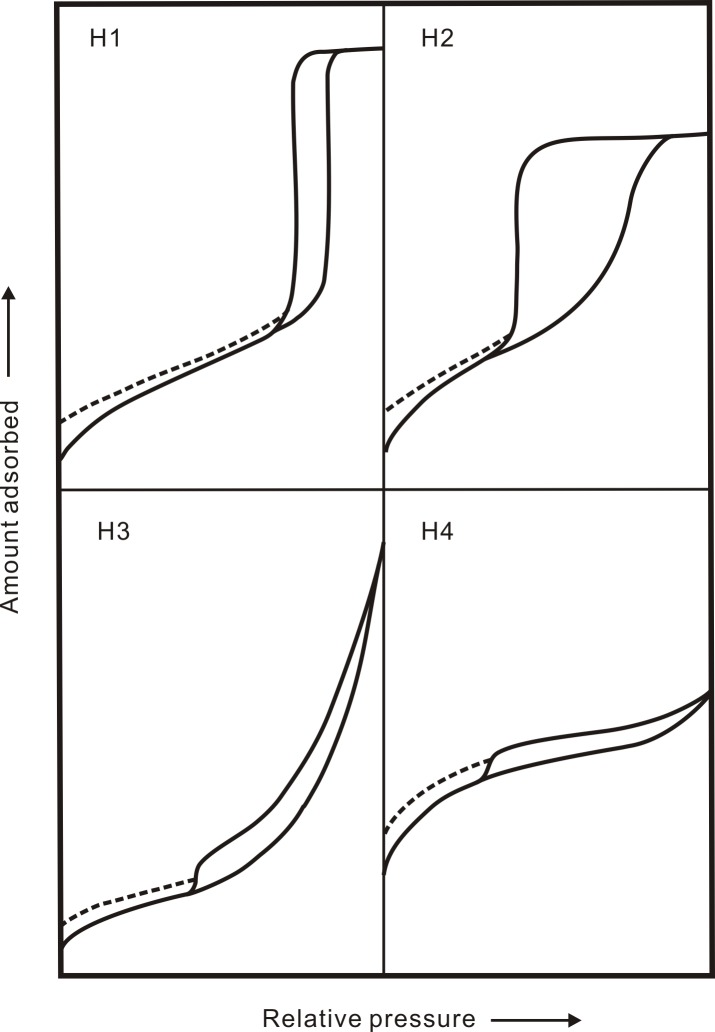
IUPAC classification of hystersis loops.

N_2_ isotherms demonstrate a wide range in adsorption and show a hysteresis pattern for all samples (Fig 1). The shape of the hysteresis loop reflects the pore geometry [20,35,44]. The adsorption isotherms show an upward trend in areas of high pressure, implying the existence of mesopores and macropores [35,45]. In the medium-pressure area, the adsorption curve does not coincide with the desorption curve, forming a hysteresis loop, indicating a capillary condensation phenomenon [35,40]. A capillary condensation phenomenon indicates the existence of open pores, e.g., ink bottle-shaped pores, slit-shaped pores and conical pores [35,44]. According to IUPAC [35], the hysteresis loop of thirty-seven samples mainly falls into two types. Samples Y-1 to Y-13 and Y-23 to Y-31 can be classified as type H2 (Fig 2; ink bottle-shaped pores), and samples Y-14 to Y-22 and Y-32 to Y-37 can be classified as type H3 (Fig 2; slit-shaped pores).

The pores in the shale of the Yanchang Formation have a mean pore size distribution between 14.66 and 17.45*Φ*, i.e., 5.59–38.63 nm, a standard deviation ranging from1.40 to 2.00, and a variation coefficient exhibiting a narrow distribution from 0.081 to 0.126. The distribution of skewness is from -1.930 to 0.302, dominated by negative contributions, implying the pore size (*D*) is smaller than the mean pore size (*D*) (#Y-1 to #Y-22 in Table 3). The mean pore size of Longmaxi shale is between 16.45 and 17.98*Φ*, i.e., 3.87–11.17 nm, with a standard deviation from 0.76 to 2.17, a variation coefficient from 0.042 to 0.175, and a skewness distribution of -3.444 to -0.698 (#Y-23 to #Y-37 in Table 3).

In accordance with the IUPAC classification [35], the pores from Yanchang shale and Longmaxi shale are mesopores. According to the principles of geological mixture empirical distribution and method of moments, the standard deviation reflects pores sorting, the variation coefficient reflects whether the pore structure is good or not, and the skewness describes whether the pore size tends toward coarse or fine [22,39]. The parameters derived from the method of moments directly reveals that the shale mean pore size (diameter) is small, the pores well sorted and the pore structure is poor. These parameters indicate poor storage and migration conditions.

## 5. Discussion

**Fig 3:**
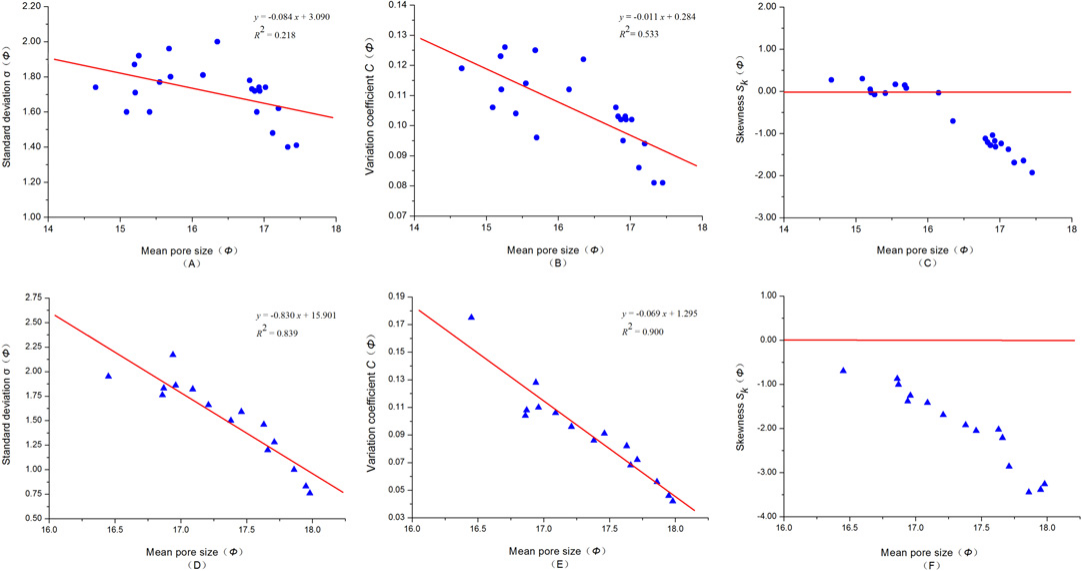
Relationship between the mean pore size (*Φ*) and standard deviation, variation coefficient and skewness. (A), (B) and (C) are relation curves of Yanchang shale from the Ordos Basin, China. (D), (E) and (F) are from Longmaxi shale in the Sichuan Basin, China.

According to the calculated characteristic parameters in Table 3, the relationship between the mean pore size (*Φ*) and standard deviation (sorting coefficient), variation coefficient and skewness were established (Fig 3). As seen from Fig 3A, the mean pore size (*Φ*) and standard deviation of Yanchang shale has a certain correlation (*R* = 0.467, *R*^2^ = 0.218): a smaller *Φ* value is associated with a larger pore size (*D*) and poorer pores sorting, it reflects the pores of larger pore size (*D*) has a large proportion of the total pores. In contrast, a larger *Φ* value, is associated with a smaller size (*D*) and better pores sorting, i.e., a large proportion of pores of smaller pore size (*D*). The mean pore size (*Φ*) of Yanchang shale shows a significant relation to the variation coefficient (*R* = 0.730, *R*^2^ = 0.533), as shown in Fig 3B. A small *Φ* value, corresponds to a large pore size (*D*), which is favorable for oil and gas storage and migration, suggesting a good pore structure (large variation coefficient). In contrast, if the *Φ* value is large, the pore size (*D*) is small, which is not favorable for oil and gas storage and migration, suggesting a poor pore structure (small variation coefficient). The skewness shown in Fig 3C extends from -1.930 to 0.302, which is dominated by the negative contribution, indicating that the pore size (*D*) is smaller than the mean value (*D*), i.e., the pore size distribution of most samples is inclined to fine skewness.

Similar to Yanchang shale, the standard deviation and variation coefficient of Longmaxi shale also showed a significant relationship with the mean pore size (*Φ*) (*R*^2^ = 0.839 in Fig 3D, *R*^2^ = 0.900 in Fig 3E). With increases in the *Φ* value, the standard deviation decreases and the same variation coefficient decreases, and vice versa. The pore size distribution of most samples is inclined to a value smaller than the mean value (*D*) (Fig 3F).

Compared with sandstone and carbonate reservoirs, shale reservoirs are tighter and, having experienced a series of diagenesis, have a stronger heterogeneity and possess more complex pore structures, with a nanoscale pore system being dominant [1,3–6,44]. According to the numerical characteristics of the geological mixture empirical distribution, the method of moments, which accounts for the diagenesis of reservoir rocks and the impact of epidiagenesis on rock pore structure [22], can be used to determine the characteristic parameters of the shale pore structure. To discuss the reasonableness of the calculated results and explore the feasibility of studying the shale pore structure using the method of moments, the calculation results have been compared with those calculated by gas adsorption theory.

N_2_ adsorption isotherms indicate a wide adsorption range and show a hysteresis loop for all samples (Fig 1). Samples Y-1-Y-13 and Y-23-Y-31 can be classified as type H2, and samples Y-14-Y-22 and Y-32-Y-37 showed type H3 (Fig 2). Yang et al. (2013, 2014) stated that pores of type H2 are favorable for gas storage but not for migration, whereas the pores of type H3, having both well-developed micropores and macropores, are favorable for gas migration due to the good pores connection [4,45]. Following this viewpoint, the standard deviation and variation coefficient of samples with type H2 are smaller than type H3. As seen from Table 3, the results of the method of moments analysis show that samples Y-1 to Y-13 and samples Y-14 to Y-22 have pore sizes (*D*) of 5.59 to 13.75 nm and 18.79 to 38.63 nm, respectively. The standard deviations for the two types of samples are 1.40 to 2.00 and 1.60 to 1.96, respectively. The former have a small pore size (*D*) and are well-sorted. The variation coefficients of 0.081 to 0.122 and 0.096 to 0.126 for the two types of samples indicate the latter samples generally have a larger value and are better for gas storage and migration. Finally, the results show the skewness of -1.930 to 0.035 and -0.076 to 0.302 for the two types of samples. Whereas samples Y-1 to Y-13 have a smaller pore size (*D*), better sorting, and a smaller variation, they are poorer for gas migration compared with Y-14 to Y-22. The characteristic parameters of the Longmaxi shale sample also indicate that the type H3 hysteresis loop of rock samples are conducive to gas storage and migration compared with type H2 samples (Table 3). Thus, the analysis results of the method of moments are consistent with the pore characteristics revealed by isotherm hysteresis loops, suggesting that the characteristic parameters derived from the method of moments can effectively reflect pore structure characteristics.

The mean pore size (*D*) derived from the method of moments is discrepant with that derived from the BJH method (Table 3). This discrepancy, may be due to the following reasons: The application of the BJH method depends on two fundamental assumptions: (1) the pores are cylindrical in shape, and (2) the physical adsorption occurs on the pore walls and capillary condensation occurs in the inner capillary volume [30]. The pore structure of shale is highly complex, with various types of pores, such as cylindrical pores, narrow slit-like pores, conical pores and ink bottle-shaped pores [20,35,45]. Shale does not have only cylindrical pores. Thus, the application of the BJH method might result in relatively large errors in the estimated pore size. Additionally, the primary purpose of the BJH method is to calculate the pore size distribution. The average pore diameter is estimated by assuming a cylindrical model [46] without considering the pores weighting within different pore size ranges. It does not conform to the actual situation in shale and makes the results less meaningful. In contrast, considering the characteristics of pore sizes from the entire range, the mean pore size calculated by the method of moments is derived from the weighted observation values and can comprehensively reflect actual shale reservoirs.

Summarizing the analysis and discussion above, the characteristic parameters calculated by the method of moments are consistent with the actual characteristics of the shale pore structure and can describe the pore structures of shale reservoir in more detail. This result suggests that studying the pore structure of shale by the method of moments is feasible and that the result is reasonable.

## 6. Conclusions

(1) The method of moments considers the influence of diagenesis and epidiagenesis on the shale pore structure, which is consistent with the geological regularity of pore size distribution and can be used to describe pore structure characteristics through various parameters quantitatively and accurately. Thus, the method of moments can be used to study the pore structure of shale.

(2) The grouping interval of shale observation values was reconstructed based on the established functional relationship between the relative pressure (*P/P*_*0*_) and mean pore size (*Φ*). By determining the observation value interval to be 11.35–18.35*Φ* and taking 0.5*Φ* as the spacing, fifteen intervals were created, and the algorithm of eigenvalues was optimized. The case study indicated that the pore structure characteristic parameters calculated by the method of moments were reasonable and reliable when compared with those derived from gas adsorption theory. This result suggests that the new method could be used to quantitatively describe pore structure characteristics.

(3) The mean pore size (*Φ*) of the shale reservoirs of the Yanchang Formation in the Ordos Basin was related to the standard deviation: a larger *Φ* value indicated better sorting, and vice versa. The mean pore size (*Φ*) was significantly related to the variation coefficient: a larger *Φ* value indicated a smaller variation coefficient and poorer pore structure, and vice versa. Similar to Yanchang shale, Longmaxi shale also exhibited these relationships.

(4) The mean pore size of Yanchang shale is 14.66–17.45*Φ*, the standard deviation distribution is 1.40–2.00, the variation coefficient ranges from 0.081–0.126 and the skewness is -1.930 to 0.302. The mean pore size of Longmaxi shale is 16.45–17.98*Φ*, the standard deviation distribution is 0.76–2.17, the variation coefficient ranges from 0.042–0.175 and the skewness is -3.444 to -0.698. Yanchang shale and Longmaxi shale both have a small mean pore size (diameter), nice pore sorting, a small pore size (diameter) degree of dispersion and fine skewness.
